# Pericardial Effusion: Rare Adverse Effect of Clozapine

**DOI:** 10.7759/cureus.4890

**Published:** 2019-06-12

**Authors:** Anandbir S Bath, Abhinav Garg, Nilanjan Gajare, Vishal Gupta

**Affiliations:** 1 Internal Medicine, Western Michigan University Homer Stryker M.D. School of Medicine, Kalamazoo, USA; 2 Psychiatry, Bronson Health, Kalamazoo, USA; 3 Cardiology, Ascension Borgess Hospital, Kalamazoo, USA

**Keywords:** pericardial effusion, clozapine, schizophrenia

## Abstract

Clozapine is a dibenzodiazepine antipsychotic used for resistant schizophrenia, which is known to be associated with side effects such as agranulocytosis, seizures, weight gain, and less commonly myocarditis/ cardiomyopathy. We present a case of a 20-year-old female who presented with chest pain, shortness of breath, and cough. She was later found to have clozapine-induced pericardial effusion that resolved after discontinuation of clozapine therapy. Our case discloses the importance to consider clozapine in the differential diagnosis of pericardial effusion as discontinuation of the drug leads to resolution of effusion, with no need for further treatment.

## Introduction

Clozapine is a dibenzodiazepine antipsychotic used in cases of schizophrenia, which are partially or fully resistant to conventional antipsychotic therapy. Clozapine is well known to be associated with side effects such as agranulocytosis, seizures, weight gain, insulin resistance, and less commonly myocarditis/cardiomyopathy [1]. Herein, a case of clozapine-induced pericardial effusion is presented, a rare side effect with only a few published case reports.

## Case presentation

**Figure 1:**
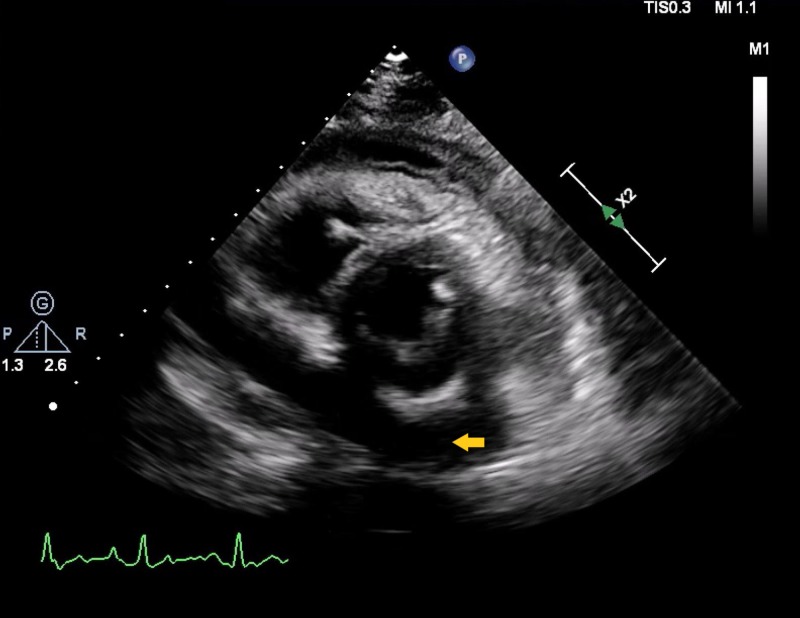
Arrow showing circumferential pericardial effusion in parasternal short axis view of transthoracic echocardiogram

A 20-year-old female presented to the emergency department with ongoing complaints of chest pain, shortness of breath, and productive cough for one week. Chest pain was sudden in onset and sharp in character. No variation with respiratory movements was noted. Medical history was significant for resistant schizophrenia on clozapine therapy. The patient denied any history of recent travel or illicit drug use. On physical examination, the patient was found to be tachycardic and tachypneic. No jugular venous distension was noted. Cardiac auscultation revealed regular S1 and S2 with no murmurs, rubs, or gallops. Laboratory investigations were found to be significant for leukocytosis with elevated C-reactive protein (CRP) of 217.9 mg/dL (reference range: 0-0.9 mg/dL) and erythrocyte sedimentation rate (ESR) of 97 mm/h (reference range: 0-29 mm/h). The respiratory infectious disease panel was negative for any viral pathogen and was pneumococcal and legionella antigen. There was no elevation noted in the procalcitonin. Rheumatological workup was also negative to delineate a cause for the effusion. CT chest revealed left lower lobe pneumonia with large pericardial effusion. The patient was started on the appropriate treatment for her community-acquired pneumonia, for which she completed seven days of therapy. Meanwhile, transthoracic echocardiogram further characterized the effusion as moderate circumferential pericardial effusion (Figure 1).

**Figure 2:**
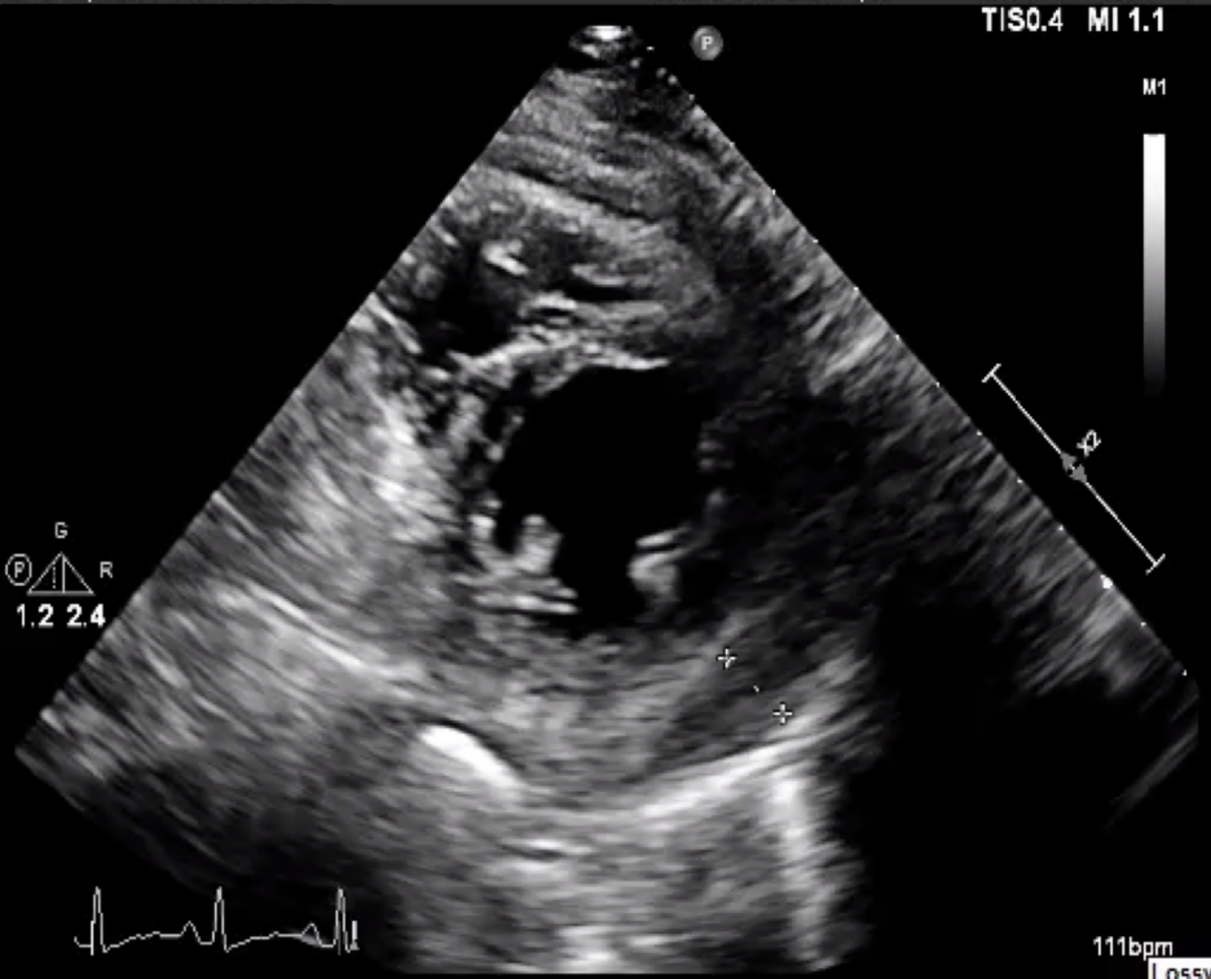
Follow-up transthoracic echocardiogram (parasternal short axis view) after three months upon discontinuation of clozapine showing resolution of the pericardial effusion

There was no evidence of tamponade physiology. Upon consultation with the patient’s psychiatrist, clozapine was stopped, as it was thought to be the cause for the patient’s non-resolving pericardial effusion. Following the discontinuation of the drug, there was a gradual improvement in the respiratory status with follow-up transthoracic echocardiogram after three months revealing a reduction in the size of pericardial effusion (Figure 2).

CRP and ESR also trended down. The patient was discharged to home with discontinuation of clozapine in a stable and improved condition.

## Discussion

Clozapine is an atypical antipsychotic used for resistant schizophrenia. It is used when patients have failed two different antipsychotics [2]. Though being more effective than typical antipsychotics, it is also associated with a hefty side effect profile, which includes agranulocytosis, seizures, central nervous system depression, myopericarditis, and cardiomyopathy. Cardiac toxicity from clozapine can be fatal as there have been reported cases of sudden cardiac death attributed to myocarditis or tamponade from pericardial effusion [3].

Polyserositis from clozapine can lead to pleural effusion, pericardial effusion, and ascites. There have been well-documented case reports of clozapine causing myocarditis and cardiomyopathy but only a few reported cases of clozapine-induced pericardial effusion. Pericardial effusion is a rare (< 1:10,000) and serious adverse effect of clozapine [4].

There is no well-documented pathophysiology behind its cardiac side effects. Postmortem myocardial biopsy in a few cases revealed eosinophilic infiltrates with myocytolysis. Peripheral eosinophilia and increased levels of IgE have also been noted, indicating the role of IgE-mediated type I hypersensitivity reaction. It can also be viewed as an acute drug reaction as pleuropericardial effusions often occur within one or two weeks of initiation of clozapine therapy [1].

Clozapine can cause pericardial effusion as early as one week after the start of treatment. The cases of delayed cardiac manifestations appearing up to months to years after the initiation of clozapine therapy have also been reported [5-7]. In our case, the patient has been taking clozapine for the past 10 months before developing significant cardiac manifestations.

The presentation of patients can range from mild viral illness to sudden death from cardiac tamponade. Patients usually have a fever with shortness of breath and pleuritic chest pain. Common physical exam findings include tachycardia and tachypnea associated with muffled heart sounds depending on the size of effusion. Troponin elevation can be seen in the scenario of myocardial injury. Inflammatory markers such as CRP and ESR are usually elevated. Suspicion for clozapine-induced pericardial effusion should be high in patients who have been on clozapine for a long time.

Since pericardial effusion is not well documented as an adverse effect, clozapine is usually overlooked as a possible etiology. Baseline ECG and echocardiography should be performed in patients with previous cardiac history who are started on clozapine therapy. All other causes such as viral infection or rheumatological conditions should be ruled out. Patients should follow-up with cardiology if diagnosed with pericardial effusion. Repeat transthoracic echocardiogram after three months of discontinuation of clozapine is recommended to ensure remission [8]. In our case, inflammatory markers CRP and ESR down trended within one week of discontinuation of clozapine therapy, and the repeat transthoracic echocardiogram after three months revealed decreased pericardial effusion.

Multiple case reports have shown that discontinuing clozapine therapy resulted in a decrease in the size of effusion [6,9]. No further treatment is indicated if clozapine is being considered as the most likely cause in the absence of definitive etiology. Follow-up ECG at three months is recommended although no official guidelines exist. There is a lack of evidence to formulate the clinical guidelines regarding clozapine rechallenge. A study showed successful rechallenge outcomes; depending upon the adverse effect clozapine was discontinued for [10].

## Conclusions

Pericardial effusion from clozapine therapy is a rare adverse effect. The timing of cardiac manifestations and initiation of treatment is variable; discontinuation of clozapine results in improvement of symptoms and resolution of pericardial effusion. No further treatment is indicated, but a follow-up echocardiogram is recommended.
